# Diagnostic value of 3.0T ^1^H MRS with choline-containing compounds ratio (∆CCC) in primary malignant hepatic tumors

**DOI:** 10.1186/s40644-016-0082-4

**Published:** 2016-08-22

**Authors:** Li Zhang, Xinming Zhao, Han Ouyang, Shuang Wang, Chunwu Zhou

**Affiliations:** Department of Diagnostic Radiology, National Cancer Center/Cancer Hospital, Chinese Academy of Medical Sciences and Peking Union Medical College, No.17, Pan Jia Yuan Nan-li, PO Box 2258, Beijing, 100021 China

**Keywords:** Liver neoplasms, Magnetic resonance image, Magnetic resonance spectroscopy, Diagnosis, Sensitivity and specificity

## Abstract

**Background:**

The purpose of this study was to investigate the diagnostic value of 3.0-T ^1^H magnetic resonance spectroscopy (^1^H MRS) in primary malignant hepatic tumors and to compare the effects of ^1^H MRS on the diagnostic accuracy of liver-occupying lesions between junior and experienced radiologists.

**Methods:**

This study included 50 healthy volunteers and 40 consecutive patients (50 lesions). Informed consent was obtained from each subject. Images were obtained on clinical whole-body 3.0-T MR system. Point -Resolved Spectroscopy was used to obtain the spectroscopy image. All conventional images were reviewed blindly by junior radiologist and experienced radiologist, respectively. The choline-containing compounds peak area (CCC-A) was measured with SAGE software, and the choline-containing compound ratio (∆CCC) was calculated. The efficacy of CCC-A and ∆CCC in the diagnosis of primary malignant hepatic tumors was determined by plotting receiver operating characteristic (ROC) curves. We also compared the effects of MRS on the diagnostic accuracy of liver-occupying lesions with junior and experienced radiologist.

**Results:**

A significant increase in mean CCC-A was observed in malignant tumors compared with benign tumors. The ROC curve showed ∆CCC had a high discriminatory ability in diagnosing primary malignant hepatic tumors with a sensitivity and specificity of 94.3 and 93.3 %, respectively. The ∆CCC area under the curve (AUC) was 0.97 that was larger than that of both junior and experienced radiologist, while the significantly statistical difference was only obtained between ∆CCC and junior radiologist (*P* = 0.01).

**Conclusion:**

^1^H MRS with ∆CCC demonstrates good efficacy in diagnosing primary malignant hepatic tumors. The technique improves the accuracy of diagnosing liver-occupying lesions, particularly for junior radiologists.

## Background

Malignant hepatic tumors, including primary liver cancer and metastatic tumors, are common worldwide. Primary liver cancer is the fifth most common malignancy in men and the eighth in women. In a 2008 survey, it was reported that there were 748,000 new cases of liver cancer diagnosed worldwide, in that year alone, with an estimated 695,000 reported deaths in the same period by the World Health Organization [1]. Moreover, the liver is the second most common site for the metastatic spread of cancer. Although progress in non-invasive imaging modalities such as ultrasound (US), computed tomography (CT) and magnetic resonance imaging (MRI) has improved the early detection and localization of malignant hepatic tumors, differential diagnosis, such as differentiating malignant from cirrhosis-related borderline lesions, still remains difficult, particularly for the junior radiologist. Biopsy is the current definitive clinical method for diagnosing dilemmatic liver lesions. However, in view of the invasiveness, costs, possible complications and sample variability of biopsy, the establishment of a simple and specific strategy to diagnose dilemmatic liver lesions are important to patient care and treatment decisions.

^1^H magnetic resonance spectroscopy (^1^H MRS) is a powerful noninvasive tool for biochemically characterizing normal and abnormal tissues [2, 3]. It has been used successfully in the evaluation of brain diseases, particularly with respect to brain tumors [4, 5]. It has also been used to distinguish between malignant and benign diseases in tissues such as the prostate [6], breast [7], and musculoskeletal system [8] . In the liver, ^1^H MRS not only has been used to evaluate liver function and diffuse hepatic disease such as liver steatosis, hepatitis and cirrhosis [9–14], but also used to distinguish between benign and malignant liver masses. Promising results have been obtained using in vitro ^1^H MRS. Soper et al. [15] performed a diagnostic correlation between ^1^H MRS and histopathology. They found that normal liver and cirrhotic liver was distinguished from hepatocellular carcinoma (HCC) with accuracies of 100 and 98 % respectively, which was consistent with Wang’s reaearch [16]. However, in vivo ^1^H MRS has been reported in only a few studies to date, and limited values were investigated. Kuo et al. [17] reported in vivo ^1^H MRS to be technically feasible, also at 3.0T, for the evaluation of focal hepatic lesions and noted limitations in distinguishing between normal liver, benign and malignant tumors. Fischbach et al. [18] suggested only a tendency towards increased choline-containing compound (CCC) levels in the spectra of HCC lesions. Overall, malignant entities did not show elevated CCC levels compared with normal liver.

To our knowledge, all these studies have compared CCC levels in tumors directly with those in normal livers. However, the liver background is highly variable [19]. Therefore, we designed a new strategy, an analysis of choline-containing compound ratio (∆CCC), to eliminate bio-variations. Thus, the purpose of our study was to prospectively investigate ∆CCC using ^1^H MRS to distinguish benign from primary malignant hepatic tumors, and to compare the effects of ^1^H MRS on the diagnostic accuracy of liver-occupying lesions between junior and experienced radiologists.

## Methods

### Study Subjects

From July 2009 to December 2010, 40 consecutive patients (mean age, 55.43 years ± 11.53 [standard deviation]; 16 women) with 50 lesions were prospectively evaluated using conventional MRI and ^1^H MRS. Inclusion criteria for our study were as follows: (a) lesions are 1 cm or more in diameter at axial images, (b) no other malignant tumor history for 5 years before the diagnosis of hepatic lesion, (c) first treatment is surgery without any preoperative treatment. Exclusion Criteria for our study were as follows: (a) Patient with unresectable tumors, (b) patients unwilling to undergo surgical treatment, (c) patients with known liver metastases, and (d) contraindications to perform MRI.

Fifty control subjects (mean age, 50.16 years ± 14.48 [standard deviation]; 21 women) followed the same MRS protocols. These control subjects had normal findings from liver imaging (MRI, US or CT) and liver function tests, and showed no clinical evidence of liver disease.

### MR Imaging

Scanning was performed with a clinical whole-body 3.0-T MR system (Signa HDxt; GE Healthcare, Chalfont St. Giles, UK) with an 8-element phased array surface coil. All subjects entered the magnet in supine feet-first position. Conventional MR plain scan was performed in all 40 patients, of which 35 patients underwent dynamic gadolinium-enhanced MRI. Point-Resolved Spectroscopy (PRESS) ^1^H MRS was performed before enhanced MRI (Table 1).

**Table 1:** Pulse sequence parameters for conventional magnetic resonance imaging and ^1^H magnetic resonance spectroscopy

	Sequence	TR (msec)	TE (msec)	Thickness (mm)	Intersection gap (mm)	Matrix	Flip angle (degree)	Scan time (sec)	Breathing
Conventional MRI									
T1-Weighted (In phase)	GRE	275	2.3	7.0	1.0	288∗192	80	19	Breath hold
T1-Weighted (Out phase)	GRE	275	5.8	7.0	1.0	288∗192	80	19	Breath hold
T2-Weighted (fat suppression)	FSE	–	102	7.0	1.0	288∗224	–	–	Respiratory-triggered
Diffusion weighted	EPI	3750	Min	7.0	1.0	128∗128	–	19	Breath hold
Dynamic enhancement^a^	LAVA-XV	2.7	1.3	3.8	–	288∗170	12	8∗6	Breath hold
MRS									
Magnetic resonance spectrograph	PRESS	1500	35	–	–	–	–	138	Intermittent breath hold
T2-weighted	SSFSE	2300	60	7.0	1.0	288∗192	–	59	Intermittent breath hold

Note: a, 35 of all 40 patients underwent this sequence

*MRI* magnetic resonance imaging, *MRS* magnetic resonance spectrograph, *GRE* gradient recalled echo, *FSE* fast spin echo, *EPI* echo planar imaging, *LAVA*-*XV* liver acquisition with volume acceleration-extended volume, *PRESS* point-resolved spectroscopy, *SSFSE* single-short fast spin echo sequences, *TR* repetition time, *TE* echo time

Conventional MR plain scans consisted of axial T1-weighted gradient recalled echo in-phase and opposed-phase sequences, an axial moderately T2-weighted fast spin-echo sequence with fat suppression, and diffusion-weighted imaging. Multiple phase dynamic enhancements scan with liver acquisition with volume acceleration-extended volume sequence as an optional sequence was performed in 35 patients.

Axial, sagittal and coronal T2-weighted single-short fast spin echo sequences were performed in each subject for ^1^H MRS localization. To maintain position consistency, an expert in abdominal radiology positioned all MRS voxels. Six saturation bands were placed around the voxel to diminish tissue contamination from the adjacent structures. In the patient group, one voxel was placed in tumor and another was placed in tumor-free tissue, respectively. The spectroscopic voxel size of tumor depends on the size of the homogeneous part of the lesion. If the lesion showed heterogeneous signal intensity, the voxel should exclude hemorrhage, necrosis and calcification. In the control group, a 2 × 2 × 2 cm^3^ voxel was placed in the right lobe of live, avoiding large vessels, bile ducts and adjacent structures. PRESS sequences were used to allow for spatial localization of the ^1^H MRS voxel. The sequences had a total of 64 acquisition times, with 2048 spectral data points at a frequency of 2500Hz and a scanning time of 138 s. Data acquisition started when the water suppression level was over 90 % and bandwidth was below 10 Hz after auto shimming. A compression belt was used in each subject. Intermittent breath-hold was adopted during data acquisition, starting at end-expiration. Based on monitoring of breathing, data acquisition could be stopped sooner or whenever the subject had to breathe again. The total acquisition time for one spectrum in the breath-hold mode amounted to 2 min 18 s on average, yielding a total measurement time of 5 to 8 min including both acquisition and resting time.

### Reading of Conventional MR Images

Conventional MR images, including plain images and dynamic gadolinium-enhanced MR images, were allocated to two radiologists, one of whom randomly chosen from a junior panel (13 radiologists with less than 5 years of gastrointestinal and hepatobiliary experience) and an experienced panel (14 radiologists with more than 10 years of gastrointestinal and hepatobiliary experience), respectively. The images were reviewed and graded blindly as either 1, 2, 3, 4 or 5 according to the following guidelines: grade 1 (definite benign lesion), grade 2 (suspected benign lesion), grade 3 (uncertain lesion), grade 4 (suspected malignant lesion) and grade 5 (definite malignant lesion).

### Analysis of MR Spectral Data

Analysis of raw spectral data was performed using commercially available software (SAGE 2005; GE Healthcare). For each patient, the data from the nearest channel was chosen. Given the ability of data processing in SAGE, we use same phase correction parameter in all frame rather than phase correction frame by frame. Spectral postprocessing included 5-Hz Gaussian filter, fast Fourier transform, and phase and baseline correction. Fully automated user-macros were developed for data post-processing. A semi-quantification method was used, and internal water (InW) was chosen as the reference metabolite to minimize the systematic and shimming variance during spectroscopy data acquisition. The peak areas at 3.2 ppm in all cases were normalized according to the peak area of the unsuppressed InW at 4.7 ppm. Using the choline-containing compounds peak area (CCC-A) in the liver ^1^H MR spectrum, we calculated the CCC-A ratio ∆CCC using the following equation:

$$ \varDelta \mathrm{C}\mathrm{C}\mathrm{C}=\left(\mathrm{C}\mathrm{C}\mathrm{C}-{\mathrm{A}}_{\mathrm{t}}\hbox{--} \mathrm{C}\mathrm{C}\mathrm{C}-{\mathrm{A}}_{\mathrm{t}\mathrm{f}}\right)/\mathrm{C}\mathrm{C}\mathrm{C}-{\mathrm{A}}_{\mathrm{t}\mathrm{f}} $$

where CCC-A_t_ and CCC-A_tf_ are the CCC-A of tumor and tumor-free tissue in the same patient, respectively.

### Statistical Analysis

The median and range of CCC-A values in the control group, benign hepatic tumors and primary malignant hepatic tumors were described. One-way ANOVA was used to compare the CCC-A among each group. The one-way *T* test was used to compare differences in the ∆CCC between benign hepatic tumors and primary malignant hepatic tumors. The diagnostic accuracies in hepatic malignant tumors of ∆CCC, conventional MRI read by junior radiologist and conventional MRI read by experienced radiologist were evaluated by receiver operating characteristic (ROC) curve analyses. The effect of MRS on the diagnostic accuracy of liver-occupying lesions was compared to junior and experienced radiologist. SPSS statistical software version 17.0 (SPSS, Inc., an IBM Company, Chicago, IL, USA) was used for all data analyses. Differences were considered significant when two-sided *P*-values were less than 0.05.

## Results and discussion

Liver ^1^H MRS was acquired in 40 patients with 50 lesions and 50 control subjects. All individuals tolerated the use of the compression belt and intermittent breath-hold. A total of 140 spectra were acquired, among which 50 spectra were localized in liver lesions, 40 in tumor-free tissue and the remaining 50 spectra were measured in normal livers of control subjects. For the 40 patients, ^1^H MRS was performed on both tumor and tumor-free tissue on the same day.

All patients were proved by surgical approach. Of 50 lesions, 15 had pathologically proven benign tumors (7 hemangiomas, 2 hydatidosises, 2 solitary necrotic nodules, 2 adenomas, 1 focal nodular hyperplasia and 1 angiomyolipoma) and the remaining 35 lesions had pathologically proven malignant tumors (26 HCC, 5 cholangiocarcinomas and 4 mixed hepatocellular and cholangiocarcinomas). The time-intervel between MRS/MRI scanning and surgery is within 2 weeks.

**Fig. 1:**
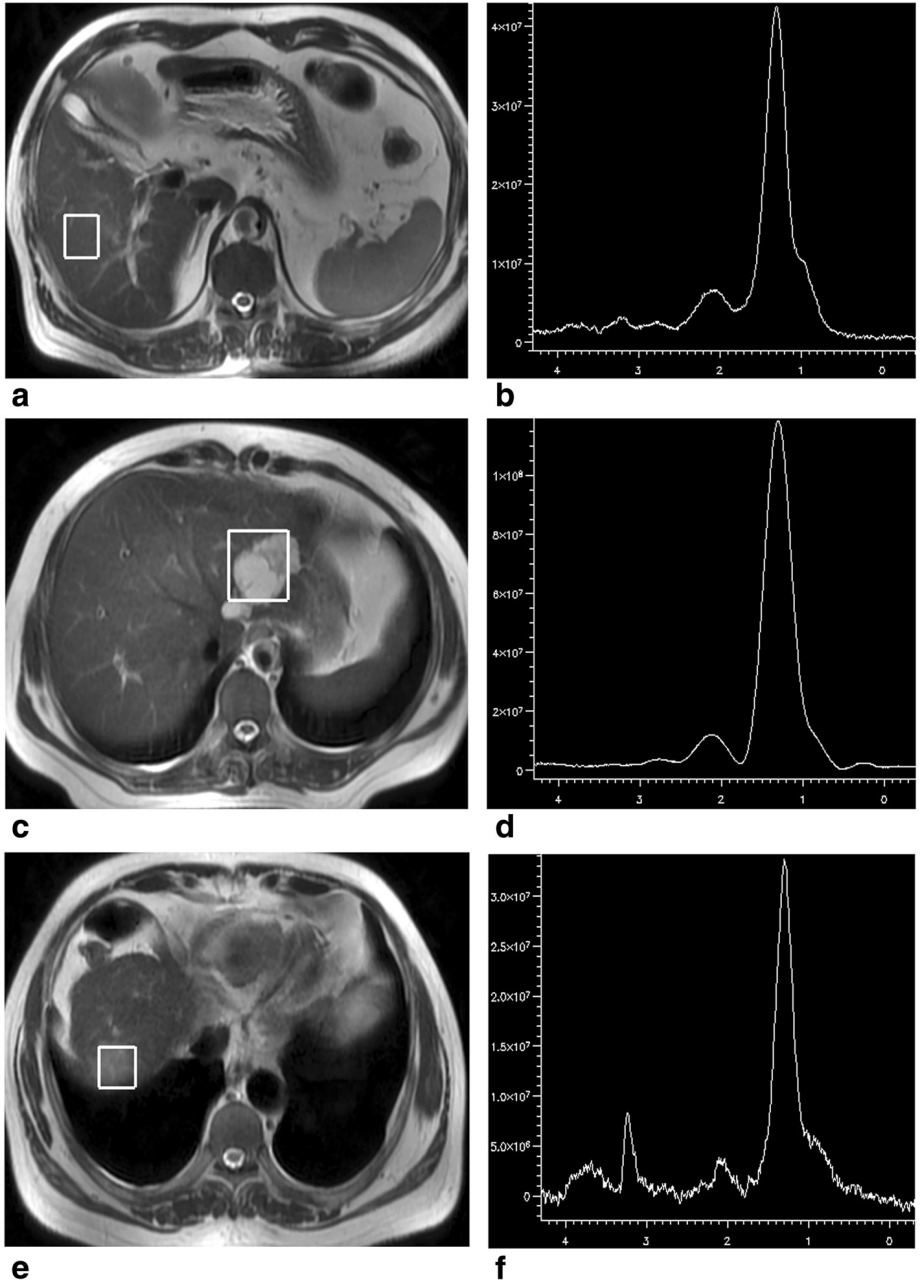
Localized magnetic resonance images and ^1^H magnetic resonance spectra in normal liver, benign tumor and malignant tumor. **a** Localized magnetic resonance image shows location of the voxel of interest in normal liver. **b** ^1^H magnetic resonance spectrum shows a choline-containing compound resonance at 3.2 ppm. **c** Localized magnetic resonance image shows location of the voxel of interest in a hemangioma. **d** ^1^H magnetic resonance spectrum shows no significant choline-containing compound resonance at 3.2 ppm. **e** Localized magnetic resonance image shows location of the voxel of interest in hepatocellular carcinoma. **f** ^1^H magnetic resonance spectrum shows a high choline-containing compound resonance at 3.2 ppm

**Fig. 2:**
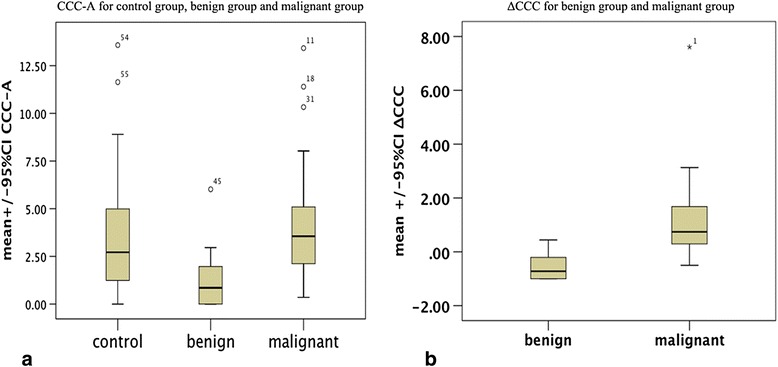
*Boxplots* show choline-containing compound and choline-containing compound ratio for study groups. **a** NO statistically significant difference of choline-containing compound exists between the control group and malignant group (*P* = 0.48). **b** Choline-containing compound ratio of malignant group is significantly higher than that of benign group (*P* < 0.01)

**Fig. 3:**
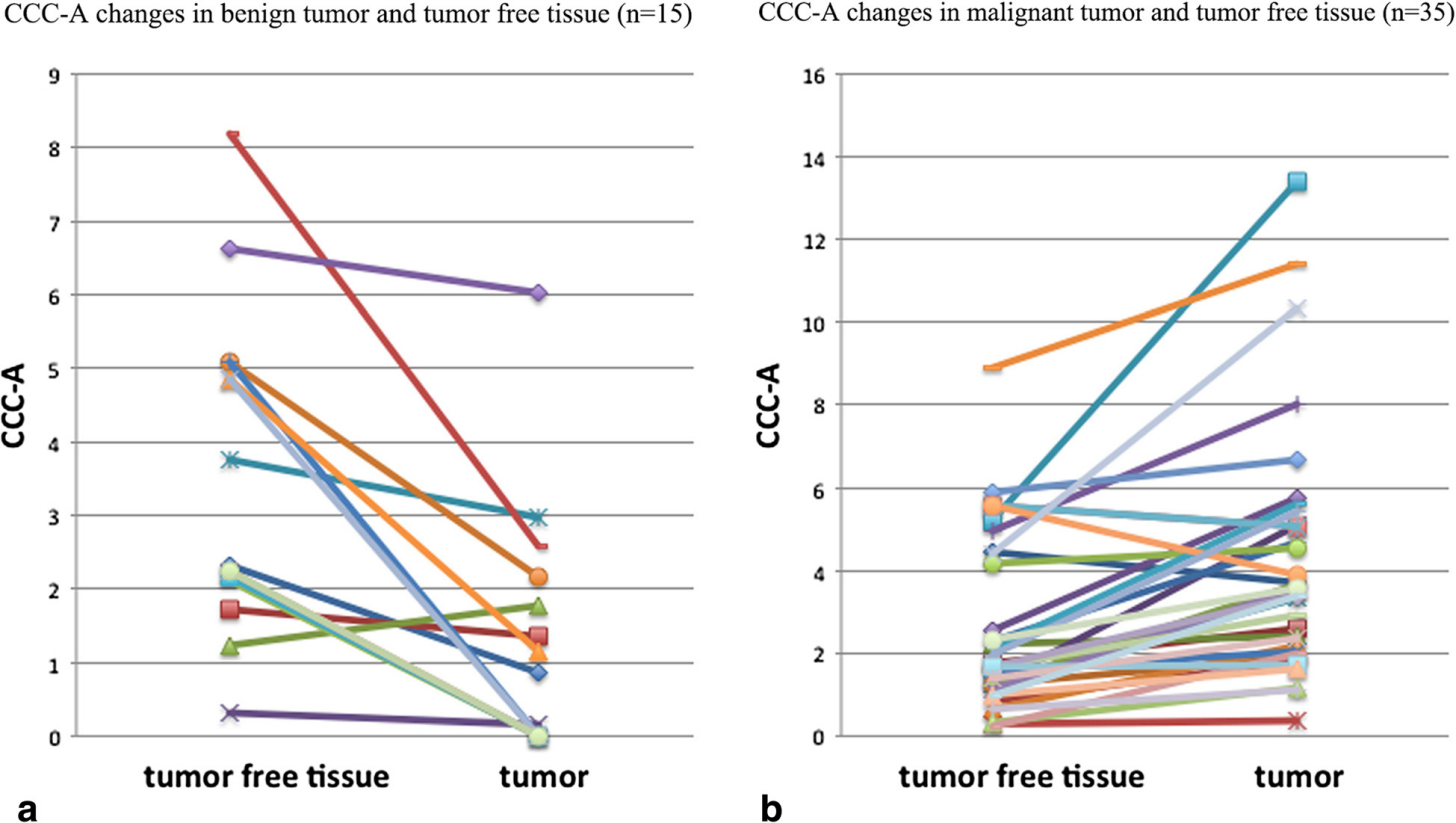
Graphs show changes of choline-containing compound between tumor and tumor-free tissue in each case. **a** Choline-containing compound changes in benign hepatic tumor and tumor-free tissue. These data show a decrease tendency. **b** Choline-containing compound changes in malignant hepatic tumor and tumor-free tissue. These data show an increase tendency

**Fig. 4:**
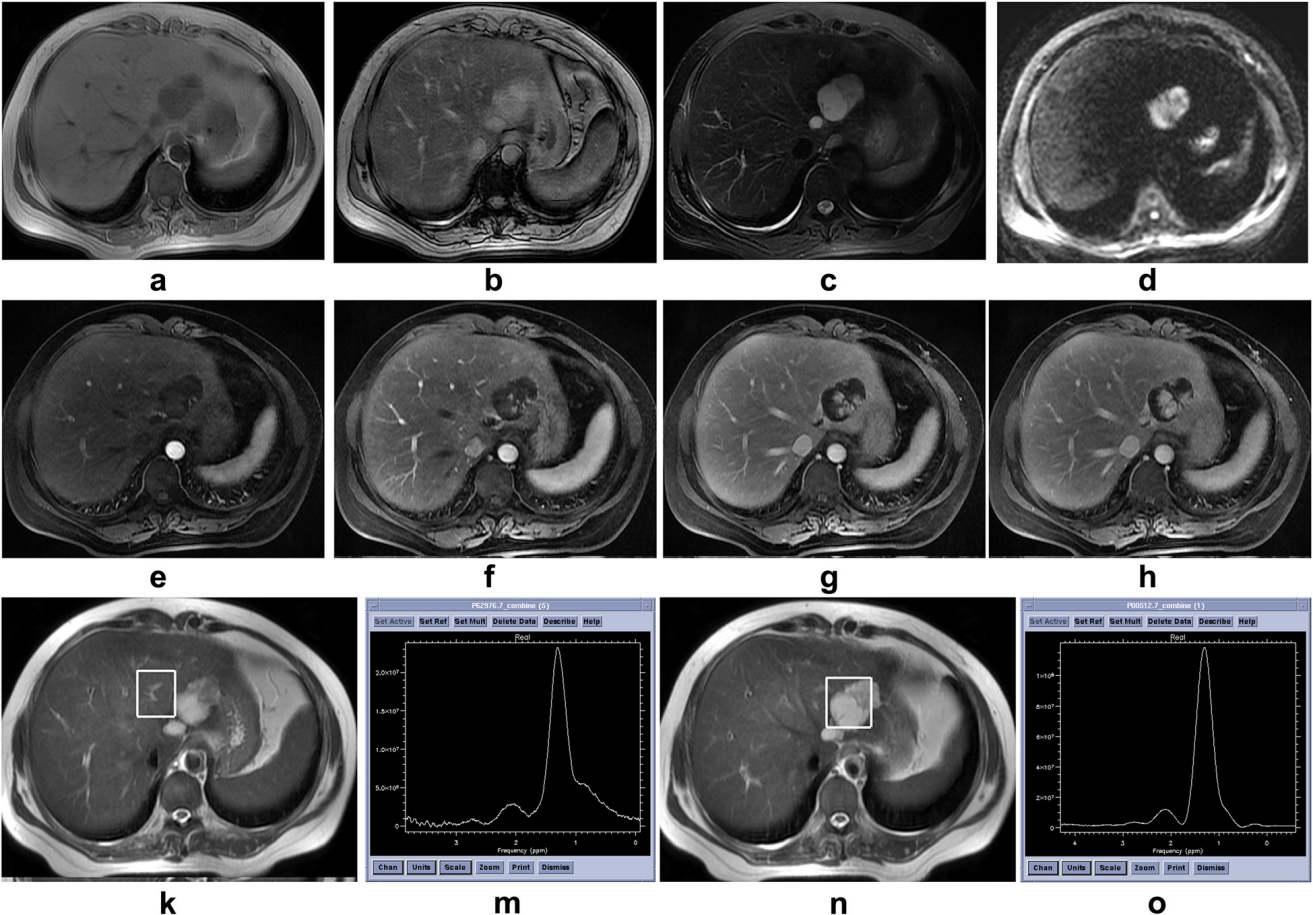
A 50-year-old female with a hemangioma in the left lobe of the liver. **a** Transverse gradient recalled echo-in phase T1-weighted magnetic resonance image shows a hypointense lesion. **b** Transverse gradient recalled echo-out phase T1-weighted magnetic resonance image shows a hyperintense lesion. **c** Transverse fast spin echo fat-saturated T2-weighted magnetic resonance image shows a hyperintense lesion. **d** Transverse spin-echo echo-planner image at b value of 600 s/mm^2^ shows high-signal-intensity lesion. **e**-**h** Multiple phase dynamic enhancement scan with liver acquisition with volume acceleration-extended volume magnetic resonance images show rapidly enhancing vessels at the periphery in the arterial phase. The lesion was then “filled- in” centripetally. **k** Localized magnetic resonance image shows location of the voxel of interest in tumor free tissue. **m** A choline-containing compound peak at 3.2 ppm was detected in tumor free tissue in ^1^H magnetic resonance spectrum, the choline-containing compound-Atf = 0.63. **n** Localized magnetic resonance image shows location of the voxel of interest in tumor. **o** No choline-containing compound peak was detected in tumor free tissue in ^1^H magnetic resonance spectrum, the choline-containing compound-At = 0

**Fig. 5:**
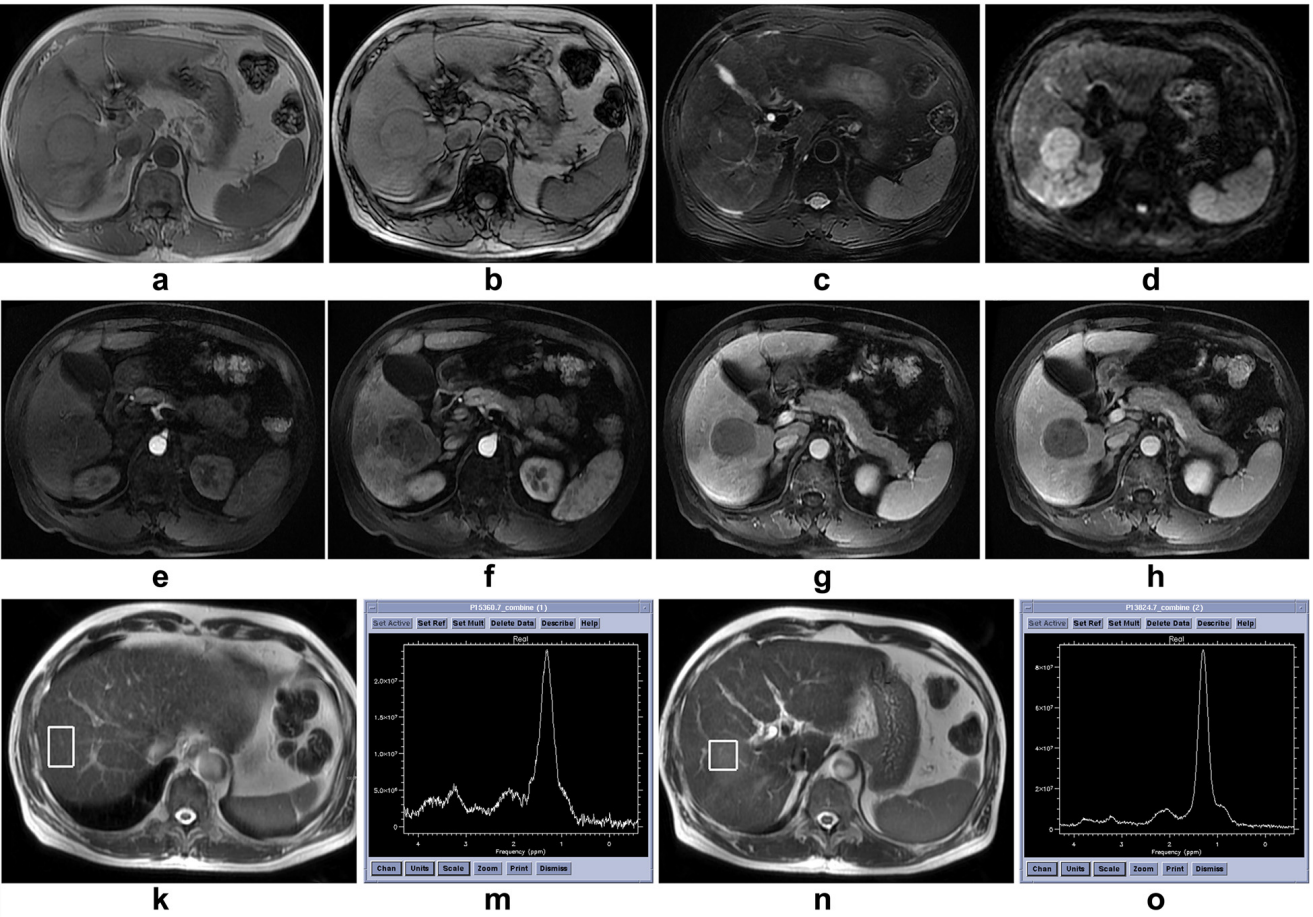
A 67-year-old male with a hepatocellular carcinoma in the right lobe of the liver. **a** Transverse gradient recalled echo-in phase T1-weighted magnetic resonance image shows an isointense tumor. **b** Transverse gradient recalled echo-out phase T1-weighted magnetic resonance image shows an isointense lesion. **c** Transverse fast spin echo fat-saturated T2-weighted magnetic resonance image shows an isointense tumor with center high-signal-intensity strip. **d** Transverse spin-echo echo-planner image at b value of 600 s/mm^2^ shows high-signal-intensity lesion. **e**-**h** Multiple phase dynamic enhancement scan with liver acquisition with volume acceleration-extended volume magnetic resonance images show no enhancement in the all phase. **k** Localized magnetic resonance image shows location of the voxel of interest in tumor free tissue. **m** A choline-containing compound peak at 3.2 ppm was detected in tumor free tissue in ^1^H magnetic resonance spectrum, the choline-containing compound-Atf = 5.89. **n** Localized magnetic resonance image shows location of the voxel of interest in tumor. **o** A choline-containing compound peak at 3.2 ppm was detected in tumor in ^1^H magnetic resonance spectrum, the choline-containing compound-At = 6.66

Based on previous research, the peak area rather than its amplitude is proportional to the amount of ^1^H nuclei in the same chemical environment and, thus, the tissue content of that chemical group or metabolite [20]. So peak area was applied as an index to evaluate the quantification of CCC in liver. A CCC resonance was detected at 3.2 ppm in all 35 malignant spectra, 9 of 15 benign spectra and 49 of 50 control spectra (Fig. 1). The mean ± 1 standard deviation CCC-A for the control group, benign tumor, and malignant tumors were 3.62 ± 2.92, 1.27 ± 1.68, and 4.16 ± 2.92, respectively (Fig. 2a). A significant increase in mean CCC-A was observed in malignant tumors compared with benign tumors (ANOVA planned contrast test, least significant difference [LSD], *P* < 0.01), and no significant statistical difference was observed between the control group and malignant tumors (ANOVA planned contrast test, LSD, *P* = 0.38 and Student-Newman-Keuls procedure, *P* = 0.48). The CCC-A of normal liver in the control group and the patients with liver lesions were 3.62 ± 2.92 and 2.83 ± 2.14, respectively. No significant difference between the two groups (*t* = −1.55, *P* = 0.124) was detected. The CCC-A changes between tumor and tumor-free tissue in the same case (Fig. 3) showed a decline among 15 benign hepatic lesions (Fig. 4) and a significant increase among 35 malignant hepatic lesions (Fig. 5). The ∆CCC (Fig. 2b) in the 35 malignant liver lesions was significantly higher than that in the 15 benign liver lesions (Table 2).

**Table 2:** Choline-containing compound value from ^1^H magnetic resonance spectroscopy between benign lesion and malignant lesion

	Benign lesion (Mean ± 1 standard deviation)	Malignant lesion (Mean ± 1 standard deviation)	*P* value
CCC-A_t_	1.27 ± 1.68	4.16 ± 2.92	<0.01
CCC-A_tf_	3.52 ± 2.19	2.53 ± 2.08	0.13
ΔCCC	−0.62 ± 0.33	1.18 ± 1.41	0.02

Note: *CCC* choline containing compounds, *CCC*-*A*_*t*_ choline-containing compounds peak area in tumor, *CCC*-*A*_*tf*_ choline-containing compounds peak area in tumor free tissue, *ΔCCC* choline-containing compounds peak area ratio

**Fig. 6:**
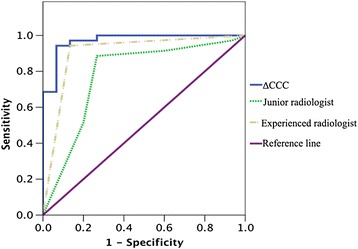
The receiver operating characteristic curves show that in vivo 1H magnetic resonance spectrum has high discriminating ability in diagnosing primary malignant hepatic tumors. The area under receiver operating characteristic curve of choline-containing compound ratio is higher than both of conventional MRI read by junior radiologist and conventional MRI read by experienced radiologist

The ROC curves for diagnosing primary malignant hepatic tumors with CCC-A and ∆CCC were plotted (Fig. 6). The area under the ROC curves (AUC) with CCC-A and ∆CCC was 0.64 (*P* = 0.03) and 0.97 (95 % confidence interval: 0.94–1.00, *P* < 0.01), respectively. If −0.09 was chosen as the cut-off value for diagnosing malignant tumors with ∆CCC, the sensitivity and specificity for malignant tumors using our criteria were 94.3 and 93.3 %, respectively. Both of the AUCs for junior and experienced radiologists were lower than the ∆CCC, while significantly statistical difference was only observed in junior radiologists compared with ∆CCC (Table 3).

**Table 3:** Receiver operating characteristic curve analysis among choline-containing compound ratio, junior radiologist and experienced radiologist

	AUC	95 % CI	*P* value
ΔCCC	0.98	0.94–1.00	0.01^a^
Conventional MRI read by junior radiologist	0.78	0.62–0.93	0.10^b^
Conventional MRI read by experienced radiologist	0.91	0.81–1.00	0.18^c^

Note: *ΔCCC* choline-containing compounds peak area ratio, *AUC* area under the receiver operating characteristic curves, *CI* confidence interval

^a^ *P* value for ΔCCC VS conventional MRI read by junior radiologist

^b^ *P* value for conventional MRI read by junior radiologist VS conventional MRI read by experienced radiologist

^c^ *P* value for ΔCCC VS conventional MRI read by experienced radiologist

According to previously published liver ^31^P MRS studies [21–23], choline is one of the components of phosphatidylcholine, an essential element of phospholipids in the cell membrane. Malignant tumors usually exhibit a high proliferation of cells and are, therefore, associated with increased metabolism of cell membrane components. This biochemical background leads to an increased presence of choline in viable cancer cells. On the contrary, necrotic tumors have a lower cell density, which leads to decreased choline concentration. Similar results were shown in liver ^1^H MRS studies, both in vitro and in vivo [15, 24], and were the foundation for evaluating the therapeutic effectiveness of the response of HCCs to locoregional therapy [25, 26]. In our study, in order to localize more precisely, the spectroscopic voxel size in the patient group ranged from 1.7 to 8 cm^3^ depending on the size of the homogeneous part of the tumor. A decrease in the voxel of interest (VOI) may decrease the signal-to-noise ratio (SNR), on the basis of previous research showing that <8 cm^3^ in VOI was insufficient for the SNR or spectral quality when using the PRESS technique in the liver [9]. Therefore, torso coil, compression belt and intermittent breath-holding techniques were used in our study to increase the sensitivity and improve the resolution of the CCC peak [27]. Another factor that may alter MR data is the choice of MR contrast material [28, 29]. Although the impact of this approach remains controversial, ^1^H MR spectra were performed before the administration of MR contrast material in all patients. For balancing signal intensity and signal contrast, an echo time (TE) of 35 ms was selected as the optimum TE, consistent with previous research [30].

Qualification of the CCC concentration is essential for characterizing differences between malignant and benign tumors. The methods of quantifying liver CCC concentrations with ^1^H MRS include the use of an internal reference and an external phantom reference [30]. Due to the high variability of liver background [19, 30], an external reference was considered to be more precise. However, this procedure, which requires accurate calibration, is extensive and therefore impractical in the clinical setting. For this reason, we selected water as the internal reference metabolite for measuring CCC-A [31].

In our research, among the three groups, CCC-A concentrations in malignant tumors achieved the highest values whereas benign tumors produced the lowest values, consistent with the report of Kuo et al. [17]. A significant difference in CCC levels between benign tumors and malignant tumors was observed in both our study and other previous studies [17, 26]. In contrast, Fischbach et al. [18] observed only a non-significant tendency for increased CCC levels in malignant tumors in their study. Several factors may have contributed to this difference. First of all, different tumor types may have resulted in a more variable CCC resonance. In a review by Podo on phospholipid metabolism (including choline) in tumors [32], it was concluded that the variation of different metabolites within tumors may be due to different tumor cell types, different phases of cell growth, and different tumor grades. In our malignant tumor group, only primary malignant hepatic tumors were included while both primary and secondary malignant hepatic tumors were observed in the research of Fischbach et al. [18] It has also been illustrated that decreased CCC levels have been observed in metastases of rectal and breast cancer [9], which may reduce the difference in mean CCC levels between malignant hepatic tumors and benign tumors. The selection of VOI may be another factor. As we know, necrosis in malignant tumors leads to a decrease in choline concentration. In our study, the VOI strictly excluded hemorrhage, necrosis and calcification in the patient group, and this may have improved the detection of CCC in tumors.

Although we observed a significant increase in CCC-A in primary malignant hepatic tumors, the ROC curve for diagnosing malignant tumors presented only a moderate value, with an AUC of 0.67. These results were highly concordant with those from the research of Kuo et al. [17], in which the AUC of ^1^H MRS in diagnosing primary malignant hepatic tumors was 0.71, with a sensitivity of 62 % and specificity of 69 %. However, in our study, the ROC curve for ∆CCC achieved a high value in diagnosing malignant tumors, with an AUC of 0.97. Individual variance of liver CCC concentrations in our population may have led to this result. Fischbach and Bruhn [9] reported that a non-significant tendency for increased CCC levels in the elderly population was observed in their study with 3.0T ^1^H MRS. The observation of no significant difference in CCC with in vivo ^1^H MRS between the control group and malignant tumors [17, 30] may prove the large variance in the population. In our study, the ∆CCC was calculated via an equation, utilizing the ratio of increased CCC levels in tumor tissue compared with tumor-free tissue in the same patient; this approach eliminates any individual variation and greatly improves the diagnostic value of ^1^H MRS in primary malignant hepatic tumors.

In our research, although both of the AUCs for conventional MRI read by junior radiologist and conventional MRI read by experienced radiologist were lower than ∆CCC, significantly statistical difference was only observed in conventional MRI read by junior radiologist compared with ∆CCC, which means that the ^1^H MRS with ΔCCC is more beneficial for junior radiologists in distinguishing between malignant and benign hepatic tumors. Conventional MR images, which have no quantitative indicators, are more rely on experience. However, junior radiologists are lack of experience and do not have profound understanding of the disease that may lead to some misdiagnosis. ∆CCC as an automatic calculated index does not need too much experience, may be a powerful assisted means in diagnosis of hepatic lesion to junior radiologists.

Our study is not without some limitations. First, CCC-A levels in tumor and tumor-free tissue were obtained from two MR spectra respectively, which was time consuming and led to possible observational management error. Hence, the development of multivoxel two- or three-dimensional chemical shift MRS would be the best method for future evaluation. Second, the scan time of 16 to 20 min was too long to be practical in clinical application. Two scans and intermittent breath holding were the main contributors. Future developments in both software and hardware are necessary to shorten the scanning procedure. Third, different tumor types should be included in future studies: our study only evaluated primary malignant tumors. Fourth, the patient population in this study was relatively small, so further studies with a larger number of patients are needed in order to reach a more robust conclusion.

## Conclusions

^1^H MRS with ∆CCC may be a useful strategy in diagnosing primary malignant hepatic tumors that, based on the findings of our preliminary study, appears to be particularly beneficial for junior radiologists. The use of an 8-element phased array surface coil, compression belt, intermittent breath-holding, and strict VOI selection, are essential for accurate metabolite measurement. Further development of MR techniques as well as additional studies with a larger number of patients and different tumor types are needed to confirm the usefulness of ∆CCC in the clinical setting.

## Abbreviations

∆CCC: Choline-containing compound ratio
^1^H MRS: ^1^H magnetic resonance spectroscopy
AUC: Area under the ROC curves
CCC: Choline-containing compound
CCC-A: Choline-containing compounds peak area
CT: Computed tomography
HCC: Hepatocellular carcinoma
InW: Internal water
LSD: Least significant difference
MRI: Magnetic resonance imaging
PRESS: Point-Resolved Spectroscopy
ROC: Receiver operating characteristic
SNR: Signal-to-noise ratio
TE: Echo time
US: Ultrasound
VOI: Voxel of interest
